# Complete genome sequence of *Saccharothrix espanaensis* DSM 44229^T^ and comparison to the other completely sequenced *Pseudonocardiaceae*

**DOI:** 10.1186/1471-2164-13-465

**Published:** 2012-09-09

**Authors:** Tina Strobel, Arwa Al-Dilaimi, Jochen Blom, Arne Gessner, Jörn Kalinowski, Marta Luzhetska, Alfred Pühler, Rafael Szczepanowski, Andreas Bechthold, Christian Rückert

**Affiliations:** 1 Department of Pharmaceutical Biology and Biotechnology, Institute of Pharmaceutical Sciences, Albert-Ludwigs-University, Freiburg, 79104, Germany; 2 Technology Platform Genomics, CeBiTec, Bielefeld University, Bielefeld, 33615, Germany; 3 Bioinformatics Resource Facility, CeBiTec, Bielefeld University, Bielefeld, 33615, Germany; 4 Senior Research Group in Genome Research of Industrial Microorganisms, CeBiTec, Bielefeld University, Bielefeld, 33615, Germany

## Abstract

**Background:**

The genus *Saccharothrix* is a representative of the family Pseudonocardiaceae, known to include producer strains of a wide variety of potent antibiotics. *Saccharothrix espanaensis* produces both saccharomicins A and B of the promising new class of heptadecaglycoside antibiotics, active against both bacteria and yeast.

**Results:**

To better assess its capabilities, the complete genome sequence of *S. espanaensis* was established. With a size of 9,360,653 bp, coding for 8,501 genes, it stands alongside other *Pseudonocardiaceae* with large genomes. Besides a predicted core genome of 810 genes shared in the family, *S. espanaensis* has a large number of accessory genes: 2,967 singletons when compared to the family, of which 1,292 have no clear orthologs in the RefSeq database. The genome analysis revealed the presence of 26 biosynthetic gene clusters potentially encoding secondary metabolites. Among them, the cluster coding for the saccharomicins could be identified.

**Conclusion:**

*S. espanaensis* is the first completely sequenced species of the genus *Saccharothrix*. The genome discloses the cluster responsible for the biosynthesis of the saccharomicins, the largest oligosaccharide antibiotic currently identified. Moreover, the genome revealed 25 additional putative secondary metabolite gene clusters further suggesting the strain’s potential for natural product synthesis.

## Background

The discovery of new antibiotics is an essential strategy to effectively combat multidrug-resistant pathogens. Two thirds of all natural products with antibiotic activity are derived from bacteria of the order *Actinomycetales*[1]. However, their potential to produce new antibiotics is not exhausted [2]. *Saccharothrix* is a genus of this order which harbors strains producing natural products of industrial interests [3]. Furthermore, *Saccharothrix* is known for its ability to glycosylate natural products hereby increasing their biological activity [4].

*Saccharothrix espanaensis* is the producer of the saccharomicins, a new class of heptadecaglycoside antibiotics [5,6] with activity against MRSA and VRE [7]. In this paper we present the classification and analysis of the genome of this capable antibiotic producer.

## Results and discussion

### Sequencing and general features of the *Saccharothrix espanaensis* DSM 44229 chromosome

The genome sequence of *S. espanaensis* was established using a whole genome shotgun approach applying next generation sequencing techniques. The initial scaffolding was performed by 454 sequencing using a 3 k paired-end library, resulting in 352 contigs in five scaffolds. In order to obtain a single scaffold, a fosmid library of 528 clones was sequenced from the ends and the sequences were mapped onto the scaffolds. This resulted in a single scaffold and delivered templates for primer walking for 283 of the 352 remaining gaps.

After fosmid and PCR-based gap closure, the chromosome was obtained as a single circular contig with a size of 9,360,653 bp. Like all completely sequenced and analyzed genomes of the *Pseudonocardiaceae* to date [8-13], the genome of *S. espanaensis* is circular with no extrachromosomal replicons, such as plasmids or chromids, detected. In total, 8,427 protein coding regions (CDS) were predicted (Table 1). The genome size and number of genes fit with the lifestyle of *S. espanaensis.* In contrast, the genomes of *Saccharomonospora viridis* and *Thermobispora bispora,* which both live under elevated temperatures in nutrient rich environments [14,15], are rather small (around 4.2 Mbp). The genomes of the *Pseudonocardiaceae* living at moderate temperatures in soil (*Actinosynnema mirum*, *Amycolatopsis mediterranei*, *Pseudonocardia dioxanivorans*, and *Saccharopolyspora erythraea*) tend to be much larger. 

**Table 1:** **General genome statistics and comparison of the completely sequenced*Pseudonocardiaceae***

**Species**	*** A. mediterranei* U 32**	*** A. mirum* DSM 43827**	*** P. dioxanivorans* CB 1190**	*** S. erythraea* NRRL 2338**	*** S. espanaensis* DSM 44229**	*** S. viridis* DSM 43017**	***T. bispora*DSM 43833**
Chromosome size	10,236,715	8,248,144	7,096,571	8,212,805	9,360,653	4,308,349	4,189,976
G + C content [%]	71.30	73.71	73.31	71.29	72.19	67.32	72.43
Chromosomal CDS	9,228	7,100	6,495	7,198	8,427	3,906	3,596
rRNA operons	4	5	3	4	4	3	3
tRNAs	52	74	47	50	80	64	63
Plasmids	0	0	3	0	0	0	0
Reference	[13]	[8]	[12]	[10]	this study	[11]	[9]

For 4,297 (51.0%) of the annotated CDS, a function could be automatically inferred using a number of sequence similarity based approaches implemented in the GenDB auto-annotator METANOR [16].

**Figure 1:**
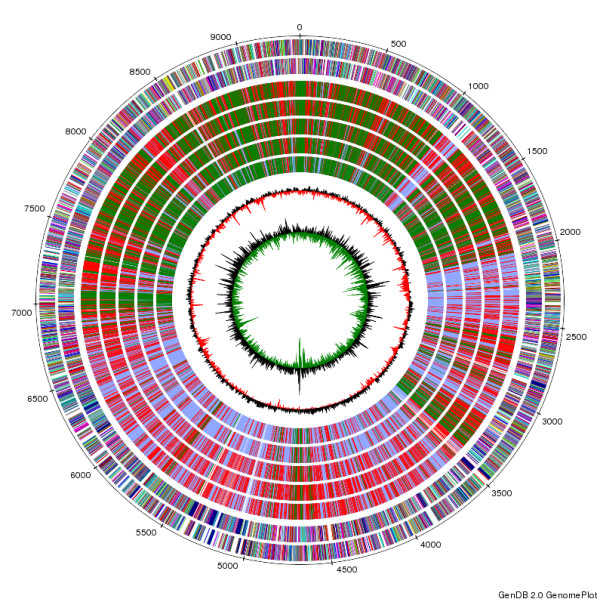
**Schematic representation of the* S. espanaensis* genome.** The genome scale is given in kilobases from the start of *dnaA*. The two outermost circles show all genes on the forward and the reverse strand, respectively, color-coded according to their predicted COG classes. The next five circles represent the genes *S. espanaensis* color-coded according to their conservation in the genomes of the other completely sequenced *Pseudonocardiaceae*. Green denotes genes present in the core genome, red those conserved at least in the two compared species and light blue indicates singletons. The comparison with *S. espanaensis* was done (from the outside in) with *A. mirum*, *A. mediterranei*, *S. erythraea*, *S. viridis* and *T. bispora*. The last two circles represent G + C content and G + C skew ((G-C)/(G + C)), both calculated for a 500 bp window with 100 bp stepping.

Based on the location of the CDS, no bias for the distribution of the genes on the leading or the lagging strand could be observed (Figure 1, outermost two circles). This coincides with no noticeable G + C skew (Figure 1, innermost circle), in contrast to the other completely sequenced *Pseudonocardiaceae*. The reason why a G + C skew is observed in some genomes while absent in others is not currently understood. The lack of a gene distribution bias and the lack of a significant G + C skew might indeed be linked. This is suggested as the other sequenced *Pseudonocardiaceae* display at least some gene distribution bias and G + C skew is thought to be due in part to a mutational bias in synonymous codons resulting in a C avoidance [17,18]. One may reasonably propose that a strong gene distribution bias might effect a faster growth rate [19]. However, as the growth rates for the completely sequenced *Pseudonocardiaceae* are not available, this remains pure speculation. 

As a first, rough classification, the amino acid sequence of all predicted CDS were compared against the eggNOG database (evolutionary genealogy of genes: Non-supervised Orthologous Groups [20]; Table 2), and the results were compared to those obtained for the other completely sequenced *Pseudonocardiaceae* (Additional file 1). This comparison revealed that the genome of *S. espanaensis* contains a relatively low number of genes involved in energy production and conversion (class C) and at only 3.66%, it is significantly below the average percentage. This is in accordance with the original description of *S. espanaensis* by Labeda *et al.*[21], who found that *S. espanaensis* cannot produce acid from most of the tested carbohydrates. Meanwhile, a disproportionally large number of genes (47.92%) could not be classified, suggesting a great potential to reveal "novel" genes. 

**Table 2:** Number of genes associated with the general eggNOG functional categories

**Code**	**Value**	**%**	**Description**
J	166	1,97	Translation
A	1	0,01	RNA processing and modification
K	475	5,64	Transcription
L	180	2,14	Replication
B	0	0,00	Chromatin structure and dynamics
D	21	0,25	Cell cycle control, cell division, chromosome partitioning
Y	0	0,00	Nuclear structure
V	112	1,33	Defense mechanisms
T	190	2,26	Signal transduction mechanisms
M	161	1,91	Cell wall/membrane/envelope biogenesis
N	0	0,00	Cell motility
Z	0	0,00	Cytoskeleton
W	0	0,00	Extracellular structures
U	27	0,32	Intracellular trafficking, secretion, and vesicular transport
O	142	1,69	Posttranslational modification, secretion, and vesicular transport
C	308	3,66	Energy production and conversion
G	213	2,53	Carbohydrate transport and metabolism
E	376	4,46	Amino acid transport and metabolism
F	86	1,02	Nucleotide transport and metabolism
H	163	1,93	Coenzyme transport and metabolism
I	167	1,98	Lipid transport and metabolism
P	242	2,87	Inorganic ion transport and metabolism
Q	171	2,03	Secondary metabolites biosynthesis, transport and catabolism
R	449	5,33	General function prediction only
S	737	8,75	Function unknown
-	4037	47,92	Not hit in eggNOG

**Figure 2:**
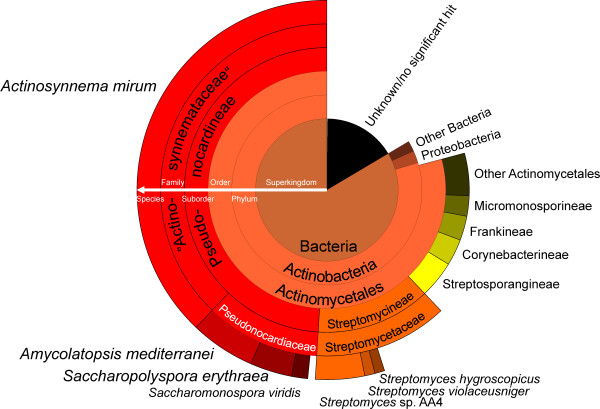
** Phylogenetic distribution of* S. espanaensis* proteins based on BlastP hits against the RefSeq database.** The amino acid sequences of all predicted CDS in the genome of *S. espanaensis* were compared against the RefSeq protein database [22] (from August 2011) using BLASTP. The species for each best hit (e-value cutoff 1e-10, hit must cover at least 75% of query and subject) was retrieved and the results were plotted from the least to the most abundantly hit group in the respective taxonomic level. For reasons of clarity, groups with few hits were either lumped together (e.g. under "Other Bacteria") or omitted entirely.

For the second general classification, a BLASTP comparison of all predicted CDS against the RefSeq database [22] was performed and the taxonomic information for the best hits was retrieved. Unsurprisingly, this analysis revealed a close relationship between *S. espanaensis* and *A. mirum* (Figure 2), currently the only other completely sequenced member [8] of the recently abolished family of the "Actinosynnemataceae" [23,24]. In the data some 38.4% of the best hits were against *A. mirum*. Almost half of the best BLASTP hits (49.7%) were against the suborder of the *Pseudonocardianeae*, while 80.7% delivered hits against proteins found in members of the order *Actinomycetales*. In general, the taxonomic distribution derived from this simple approach matches the phylogenetic distance derived from the 16S rDNA (Additional file 2), although it does not take into account the variable genome sizes. 15.2% of the CDS delivered no significant BLASTP hits against the RefSeq database, which is well within the range (10.5% - 19.0%) of the other completely sequenced *Pseudonocardiaceae*. 

### Comparison of the *S. espanaensis* genome with other completely sequenced *Pseudonocardiaceae*

**Figure 3:**
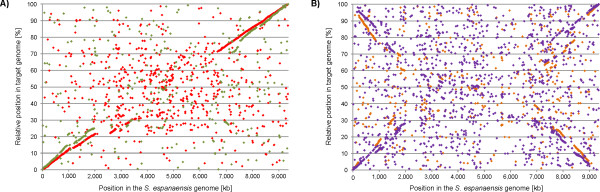
**Whole genome comparison of* S. espanaensis.*** To analyze gene synteny, the amino acid sequences of all predicted CDS in the genome of *S. espanaensis* were compared against those of (**A**) *A. mirum* (red) and *S. erythraea* (green) as well as *A. mediterranei* (purple) and *S. viridis* (orange) using the bidirectional BLAST comparison implemented in EDGAR. Aligning all genomes at *dnaA*, the position of each potential ortholog was then plotted against the position in the *S. espanaensis* genome. In order to accommodate different genome sizes, the relative position is used for the target genomes.

For *A. mediterranei* and *S. erythraea *[10,13] it was observed that there exists a striking bias concerning gene conservation and synteny. In both cases, the genes conserved between *A. mediterranei* respectively *S. erythraea* and other *Actinomycetales* were found to be preferentially located close to the *oriC* and at least some synteny could be detected. With six genomes of the *Pseudonocardiaceae* available, we performed a similar analysis using EDGAR. Indeed, a strong correlation between conservation and synteny around the *oriC* could be observed, especially when comparing *S. espanaensis* with *A. mirum* or *S. erythraea* (Figure 3A), but also for the others (Figure 3B: *S. viridis* and *A. mediterranei*). This also holds true if one of the other six genomes is used as a basis (data not shown, available via the open EDGAR project). A similar observation was made for the genomes of the species *Streptomyces *[25,26] where the conserved core is located in the middle of the circular genome while the accessory genes are found at the variable ends. Interestingly, in another genus of the *Actinomycetales* with circular genomes, *Corynebacterium*, this positional bias is not observed [27]. 

**Figure 4:**
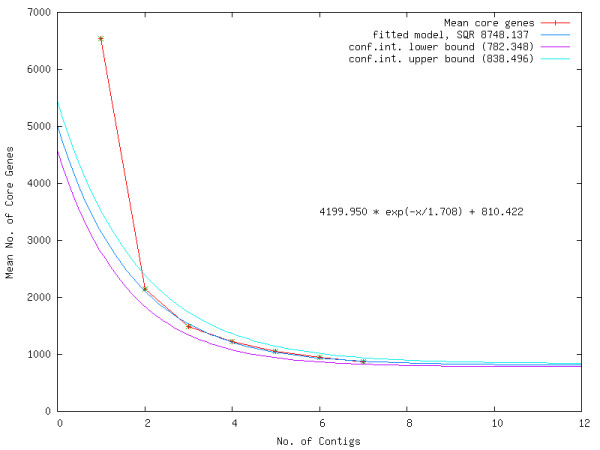
**Development of the core genome of the* Pseudonocardiaceae.*** Using EDGAR, the development of the core genome of the *Pseudonocardiaceae* was extrapolated by calculating the mean core genome numbers for all possible permutations of genomes (red crosses/line). By non-linear least squares curve fitting, an exponential decay function (dark blue curve and equation) was fitted to the mean core data. A 95% confidence interval was calculated for the fitted model, and the boundaries are displayed (light blue and purple curves). Using the genomes of *A. mirum*, *A. mediterranei*, *P. dioxanivorans*, *S. erythraea*, *S. espanaensis*, *S. viridis*, and *T. bispora*, a final core genome of approximately 810 genes is predicted, with the current core of the seven analyzed species consisting of 864 genes.

This raised the question whether this degree of conservation and synteny is more pronounced if several species are taken into account, i.e. for the genes making up the core genome of the family. As a first step to answer this question, the family core genome was calculated, once again using EDGAR. In a first step the core genome was calculated for every possible unique subset of the seven genomes. Following this, an exponential decay function was fitted to the observed core genome counts for every genome quantity as described by Tettelin *et al. *[28]. Using this function, the development of the core given a presumed sequential addition of more genomes was extrapolated. This led to the predicted core genome of about 810 genes (Figure 4), i.e. the theoretical core of all *Pseudonocardiaceae*. With the core genome of these seven species comprised of 864 genes the 810 value is almost reached with the seven genomes used. A test including two *Streptomyces* species (*S. coelicolor* and *S. avermitilis*) only reduced the number of genes in the resulting core by about 45 genes to 765 which is already reached with the then nine genomes (data not shown, available via the open EDGAR project). This is quite surprising, as *Streptomyces* species usually possess linear ends. This implies that *Pseudonocardiaceae* and *Streptomycetaceae*, despite having different chromosome topologies, retain their core genes close to the *oriC*. 

This implication is backed by further analyzing the correlation between gene conservation and location relative to the *oriC*: the core genes are found predominantly clustered around the *oriC* (Figure 1, genes depicted in green in circles 3–7) while genes conserved in only some species and the 2,967 *S. espanaensis* singletons (Figure 1, genes depicted in red respectively light blue in circles 3–7) are found farther away from the *oriC*.

### The genes of the accessory genome of *S. espanaensis* are either ancient and/or obtained from closely related species

**Figure 5:**
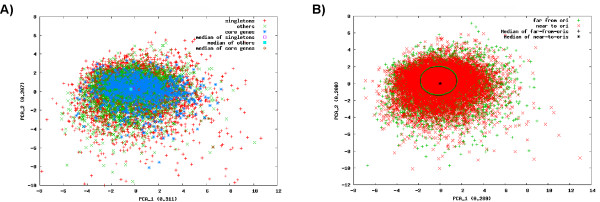
**Principle component analysis of the dinucleotide frequencies of the* S. espanaensis* CDS. A**) Using EDGAR, all CDS from *S. espanaensis* were divided into three groups: "core" (conserved in all six completely sequenced *Pseudonocardiaceae*; blue "*"), "other" (shared between *S. espanaensis* and at least one other *Pseudonocardiaceae* species; green "x") and singletons ("unique" in *S. espanaensis*; red " + "). For all genes the relative dinucleotide frequencies were calculated, a PCA was performed using the R package and the results for the two main components are plotted. In addition, the median values for all three distributions were calculated and plotted. (**B**) Using the same calculation as in A, the genes were divided in relation to their position in the genome relative to the origin of replication. Genes close to the *oriC* (corresponding to the "top half" of the genome) are given as red "x", genes closer to the terminus ("bottom half" of the genome) are depicted as green " + ". Median points are denoted as black "*" and " + ", green and black circles mark the 90% boundaries.

The distribution of core and accessory genes in the genome raises the question of what mechanism is causing this peculiar pattern. As multiple independent circularization events are exceedingly unlikely, the genome of the common ancestor of the *Pseudonocardiaceae* was either extremely large and suffered gene loss during speciation or it was rather small and most of the accessory genes were acquired later. This in turn raises the question of whether there is evidence that the accessory genes were acquired recently. To shed light on this timing, we performed a principle component analysis (PCA) of the relative polynucleotide frequencies of all of the genes. Analysis of polynucleotide frequencies were shown to be useful for classification of short DNA fragments obtained from different genomes [29]. In our case, no significant differences on the dinucleotide level could be observed for most of the genes, neither when comparing core and accessory genes (Figure 5A), nor when comparing genes close and distant to the *oriC* (Figure 5B). We also found that this held true for the tri- and tetranucleotide frequencies (data not shown). Therefore, the accessory genes present in the genome of *S. espanaensis* had either time to "adapt" to the prevalent polynucleotide bias or were obtained from genomes with a similar polynucleotide composition. While the currently available data does not suffice to decide which scenario is more likely, this question should be revisited when more *Pseudonocardiaceae* genomes become available. 

### Genes participating in the synthesis of saccharomicins

**Figure 6:**
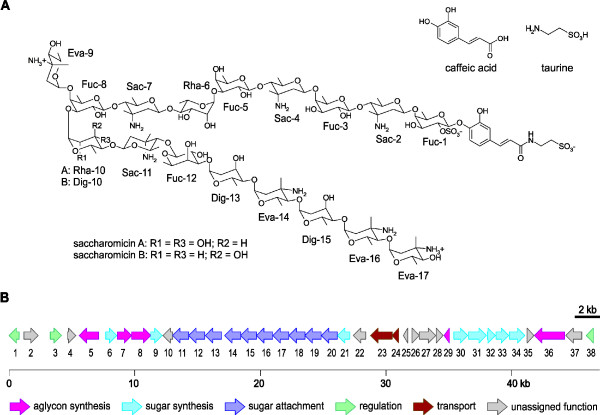
**The saccharomicin gene cluster from* S. espanaensis.*** (**A**) Chemical structures of caffeic acid, taurine, saccharomicin A and B. Fuc, d-fucose; Sac, d-saccharosamine; Rha, l-rhamnose; Eva, l-4-epivancosamine; Dig, l-digitoxose [6]. (**B**) Organization of the saccharomicin cluster. Proposed functions for individual CDS are summarized in Additional file 1.

The most relevant accessory genes from the medical point of view are those for the production of secondary metabolites. For example, *S. espanaensis* is known to produce the saccharomicins A and B. Due to their activity against multi-resistant pathogens, these two compounds comprise a promising class of new antibiotics [6]. They possess an interesting chemical structure consisting of an exceptional N-(3,4-dihydroxycinnamoyl) taurine aglycon and a heptadecaglycoside side chain (Figure 6A) [6]. Previously, Berner *et al. *[30] were able to elucidate part of the saccharomicin biosynthetic pathway using a cosmid library. They reconstructed one half of the aglycon by performing co-expression of *sam8* and *sam5*. These genes, encoding a tyrosine ammonia-lyase and a 4-coumarate 3-hydroxylase, respectively, convert l-tyrosine via trans-p-coumaric acid to caffeic acid. 

Our analysis of the complete genome sequence of *S. espanaensis* revealed the best candidate for the saccharomicin biosynthetic gene cluster (sam) (Figure 6B and Additional file 3), because this cluster harbors both the genes responsible for the production of the caffeic acid moiety of the aglycon, as well as ten glycosyltransferase genes necessary for the formation of the oligosaccharide side chain. The sam-cluster does not belong to the core genome and is located at the lower part of the circular chromosome, close to termination of replication. It comprises approximately 47,000 base pairs and is predicted to encode 38 genes. The identification of the sam-cluster allows further insights into the assembly of these new antibiotics. The product of *sam7* shows similarity to acyl-CoA synthetases. It is therefore tempting to speculate that Sam7 may be involved in the synthesis of caffeoyl-CoA, which might be used to link taurine to form the entire aglycon. This reaction may be catalyzed by Sam36 which shows similarities to penicillin amidases. However, the synthesis of taurine has not been described in bacteria. In mammals, taurine originates from l-cysteine via cysteine sulfinic acid. Even though the dioxygenation of cysteine has been shown in bacteria [31], they are not able to produce taurine due to the lack of a cysteine sulfinic acid decarboxylase (csad). The same is true for *S. espanaensis*: there are potential cysteine dioxygenase genes (Ses72720, Ses71790) but no csad homologues are detectable in the genome. However, *sam29* may encode the required chemistry. The protein Sam29 shows similarities to aspartate-1-decarboxylases which are responsible for the decarboxylation of l-aspartate forming β-alanine. The structures of cysteine sulfinic acid and l-aspartate are identical with the exception of one atom. While aspartate possesses a carbon as part of the carboxyl group, cysteine sulfinic acid contains sulfur in this position. As a result of this similarity we suggest that Sam29 might be able to decarboxylate cysteine sulfinic acid. If this is found to be correct, *sam29* would represent the first gene responsible for the production of taurine in bacteria.

In addition to genes accountable for the formation of the aglycon, there are candidates encoding proteins involved in sugar synthesis and attachment. To link the 17 sugars to the aglycon, there are ten glycosyltransferase genes (*sam11*-*sam20*) in the cluster. Consequently, some of the glycosyltransferases may work iteratively. Because sequence analyses of those genes gave no further indication, the exact function of each glycosyltransferase will have to be experimentally investigated.

### Potential for secondary metabolite production

**Figure 7:**
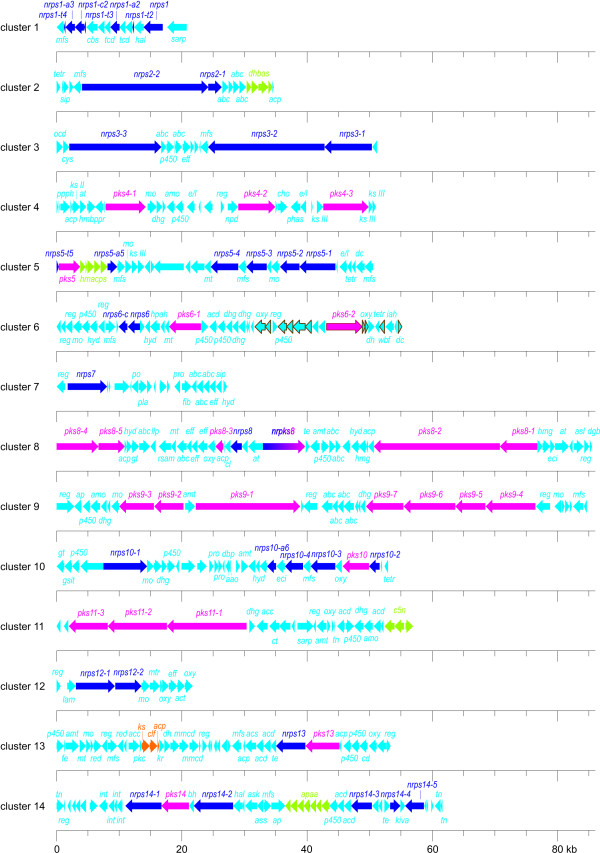
*** S. espanaensis* gene clusters for nonribosomal peptide and polyketide biosynthesis.** Genes encoding nonribosomal peptide synthases are depicted in dark blue, type I polyketide synthases in red and type II polyketide synthases in orange. The genes involved in the synthesis of putative precursors are highlighted in light green. The remaining genes of the clusters are presented in pale blue. All genes involved in the biosynthesis of an enediyne core in cluster 6 are framed brown. *aao*, l-amino-acid oxidase; *abc*, ABC transporter; *acc*, acyl-CoA carboxylase; *acd*, acyl-CoA dehydrogenase; *acp*, acyl carrier protein; *acs*, acyl-CoA synthetase; *act*, acyl-CoA transferase; *amo*, amine oxidase; *amt*, aminotransferase; *ap*, aminopeptidase; *ask*, adenylylsulfate kinase; *asl*, AMP-dependent synthetase and ligase; *ass*, sulfate adenylyltransferase; *at*, acyl transferase; *bh*, beta-hydroxylase; *cbs*, carbamoyltransferase; *cd*, cysteine desulfurase; *cho*, cholesterol oxidase; *cl*, chlorinating protein; *clf*, chain length factor; *ct*, carboxyltransferase; *cys*, cysteine synthase; *dbp*, DNA-binding protein; *dc*, decarboxylase; *dgb*, glyoxalase/bleomycin resistance protein/dioxygenase; *dh*, dehydratase; *dhbas*, protein involved in the synthesis of activated 2,3-dihydroxybenzoic acid; *dhg*, dehydrogenase; *e/l*, esterase/lipase; *eci*, enoyl-CoA hydratase/isomerase; *eff*, efflux protein; *gsit*, glutamine—scyllo-inositol transaminase; *gt*, glycosyltransferase; *hal*, histidine ammonia-lyase; *hmacps*, protein involved in the synthesis of hydroxymalonyl-ACP; *hmbppr*, 4-hydroxy-3-methylbut-2-enyl diphosphate reductase; *hmg*, hydroxymethylglutaryl-CoA synthase; *hpah*, 4-hydroxyphenylacetate-3-hydroxylase; *hyd*, hydrolase; *int*, integrase; *kr*, ketoreductase; *ks II*, FabF-like protein; *ks III*, FabH-like protein; *lam*, lysine 2,3-aminomutase like protein; *llp*, lipolytic protein; *lys*, protein involved in lysine synthesis via alpha-aminoadipate; *mfs*, transporter of the major facilitator superfamily; *mmcd*, methylmalonyl-CoA decarboxylase; *mo*, monooxygenase; *mt*, methyltransferase; *mtr*, methionyl-tRNA synthetase; *npd*, 2-nitropropane dioxygenase; *ocd*, ornithine cyclodeaminase; *oxy*, oxidoreductase; *p450*, cytochrome P450; *phas*, polyhydroxy alkanoic acid synthase; *pkc*, polyketide cyclase; *ppph*, 2-polyprenylphenol 6-hydroxylase; *pro*, protease; *reg*, regulatory protein; *rsam*, radical SAM protein; *sarp*, streptomyces antibiotic regulatory protein; *sip*, siderophore-interacting protein; *tcd*, taurine catabolism dioxygenase; *te*, thioesterase; *tetr*, protein similar to the tetracycline repressor; *tk*, transketolase; *tn*, transposase.

Aside from the antibiotics saccharomicin A and B, no further secondary metabolites had been known to be produced by *S. espanaensis* before genome sequencing. The secondary metabolites search tool antiSMASH [32] identified 31 putative clusters, including the sam-cluster. However, several inaccuracies by the search tool had to be manually curated. This resulted in the total number of 26 clusters potentially producing secondary metabolites (Table 3, Figure 7, and Additional file 4). All secondary metabolite clusters are located outside the core genome of *S. espanaensis*. 

**Table 3:** **Secondary metabolite cluster comparison of the completely sequenced*Pseudonocardiaceae***

**Species**	**Terpene**	**PKS**	**NRPS**	**Hybrid**	**Other**	**Total**
* A. mirum*** DSM 43827**	4	10	3	5	1	**23**
*A. mediterranei*** U32**	4	6	7	4	4	**25**
*P. dioxanivorans*** CB1190**	2	0	1	0	2	**5**
*S. erythraea*** NRRL 2338**	10	11	4	2	3	**30**
*S. espanaensis*** DSM 44229**	7	3	5	6	5	**26**
*S. viridis*** DSM 43017**	2	2	2	0	2	**8**
*T. bispora*** DSM 43833**	1	1	0	0	3	**5**

Seven clusters possibly encoding terpenoid biosynthetic enzymes are distributed throughout the genome. The C5-precursors required for their biosynthesis originate from the methylerythriol phosphate pathway, for which all genes are present. Terpene derived metabolites include carotenoid pigments (crt) serving as UV protector and odorous substances providing actinomycetes with their characteristic smell [33,34].

In the genome a total of five nonribosomal peptide synthetases (NRPS) encoding clusters could be identified (cluster 1, 2, 3, 7 and 12). Common metabolites produced by NRPS in actinomycetes are for example the antibiotic vancomycin, the cytotoxic agent bleomycin and the iron-scavenging siderophore griseobactin [35-37]. The proteins producing such polypeptides are usually composed of modules consisting of condensation, adenylation and thiolation domains. Remarkably, cluster 1 possesses a set of genes which code for only one *nrps* domain each. Consequently, this small cluster of about 20 kb may harbor an archetype of *nrps*. These genes, consisting of a condensation, an adenylation and a thiolation domain, respectively, might be ancestors of our contemporary *nrps* genes composed of a chain of different domains. The modules of all *nrps* genes and the specificity of their adenylation domains are listed in Additional file 5.

In addition to the clusters producing nonribosomal peptides, there are three clusters producing type I polyketides (cluster 4, 9 and 11). These natural products are synthesized by decarboxylative condensation of malonyl-CoA derived extender units. Polyketide synthetases (PKS) possess as well a modular assembly and are the well-known producers of the antibiotic erythromycin and the immunosuppressant tacrolimus in other actinomycetes strains [38,39]. The modules of all polyketide synthetases identified in *S. espanaensis* are listed in Additional file 6. The PKS containing clusters are the largest in the genome of *S. espanaensis* and comprise between 50 and 86 kb.

Besides pure NRPS or PKS clusters, we identified six clusters which harbor both types of these secondary metabolite synthesis genes (cluster 5, 6, 8, 10, 13 and 14). Among them, cluster 6 shows high similarity to the maduropeptin cluster from *Actinomadura madurae* ATCC 39144 [40]. Consequently, cluster 6 is identified as a putative enediyne cluster.

Cluster 13 is not only a NRPS/PKS type I hybrid, but it also contains genes coding for type II PKS. The type II PKS part of the cluster is highly similar to the kinamycin gene cluster from *Streptomyces murayamaensis* [GenBank:AH012623.1]. All core and tailoring enzymes required for the production of kinamycin are present in cluster 13. Additionally it possesses further genes encoding tailoring enzymes like a P450 dependent monooxygenase (*ses54730*), an aminotransferase (*ses54750*) and a methyltransferase (*ses54760*). The type I PKS and the NRPS part of the cluster may contribute to the modification of the core structure of cluster 13, leading to a kinamycin derivative. Another hybrid, Cluster 14, shows similarities to the azinomycin B biosynthetic gene cluster from *Streptomyces sahachiroi *[41]. Azinomycin B is an antitumor agent consisting of a PKS-derived naphthoate attached to a nonribosomal peptide. The peptide part of the molecule is composed of unusual building blocks like α-ketoisovaleric acid and an aziridino-[1,2a]-pyrrolidinyl amino acid. Cluster 14 harbors *ses56840*, whose gene product is similar to AziC1 responsible for the production of α-ketoisovaleric acid from valine. Additionally, all genes are present for the production of the aziridino-[1,2a]-pyrrolidinyl amino acid (*aziC2aziC10*; *ses56710*, *ses56680*, *ses56700*, *ses56740*, *ses56730*, *ses56750*, *ses56760*, and *ses56690*) except for a homolog to *aziC8*. Furthermore, the cluster possesses homologues of *aziD2* and *aziD3* (*ses56770* and *ses56780*) which are responsible for tailoring modifications of the molecule. However, the PKS modules of the azinomycin B cluster differ from the ones found in cluster 14. Therefore we propose that the PKS derived moiety of the compound produced by cluster 14 is not a naphthoate moiety as in azinomycin B. Another interesting fact is that cluster 14 is flanked by transposase and integrase genes. This suggests that the cluster was probably introduced into the genome of *S. espanaensis* by horizontal gene transfer.

In addition to the secondary metabolite gene clusters belonging to the prevalent groups of NRPS, PKS and terpene synthases, the genome of *S. espanaensis* also contains rare types of clusters: it harbors a putative aminocyclitol cluster, a melanin cluster and two putative lantibiotic clusters (lan1 and lan2). Lantibiotics are ribosomally produced and posttranslationally modified polypeptides which contain thioether-cross-linked amino acids [42].

## Conclusion

The complete genome of *S. espanaensis* was sequenced and compared to the genomes of the other completely sequenced *Pseudonocardiaceae*. Thereby, the expected core genome of the family could be predicted to consist of about 810 genes. While the origin of the accessory genome of *S. espanaensis* remains unclear, some evidence provided suggests that the accessory genes are either part of the genome for quite some time and/or were obtained from bacteria with a similar polynucleotide composition.

Besides providing some insights into the genome evolution of the *Pseudonocardiaceae*, the genome sequence delivered a good candidate cluster for the production of the saccharomicins. The newly identified sam-cluster consists of 38 genes and comprises approximately 47,000 base pairs. It harbors a presumed operon of ten glycosyltransferase genes centered in the cluster. To our knowledge, there is no other biosynthetic gene cluster identified to date which includes this high number of glycosyltransferase genes. Nevertheless, to produce the heptadecasaccharide side chain of the saccharomicins even more than ten glycosylation steps are proposed, so some glycosyltransferases should work iteratively. We anticipate that the complete genome sequence will facilitate the production and modification of the saccharomicins, by either improving precursor supply or by engineering of genes belonging to the cluster to obtain novel variants.

## Methods

### Pyrosequencing of *Saccharothrix espanaensis* DSM 44229^T^

The type strain of *Saccharothrix espanaensis* (DSM 44229) was obtained as lyophilized culture from DSMZ (Braunschweig, Germany). Genomic DNA was isolated from 30 ml cultures grown in tryptone soy broth (TSB) [43] at 28°C for 24 hours. Total DNA isolation was performed according to the salting out procedure followed by RNase treatment [43].

10 μg was used to construct both a 3 k PE and a WGS library for the pyrosequencing on a Genome Sequencer FLX (Roche Applied Science). Assembly of the shotgun reads was performed with the GS Assembler software (version 2.0.00.22). A total of 1,536,941 reads (404,849,780 bp) was assembled into 352 contigs in five scaffolds.

### Completion of the draft sequence

For gap closure and assembly validation, the genomic contigs were bridged by (i) a fosmid library of 528 clones spanning all but 69 gaps and (ii) 69 PCR products addressing the remaining gaps. For finishing of the genome sequence, the CONSED software package [44] was used.

Sequencing of fosmid ends was carried out by IIT GmbH (Bielefeld, Germany). Gaps between contigs of the whole genome shotgun assembly were closed by sequencing on PCR products and fosmid clones carried out by IIT GmbH on ABI 377 sequencing machines. To obtain a high quality genome sequence, all bases of the consensus sequence were polished to at least phred40 quality by primer walking. Collectively, 854 sequencing reads were added to the shotgun assembly for finishing and polishing of the genomic sequence.

### Genome analysis and annotation

In a first step, gene finding was done by using GISMO [45] and an automatic annotation was performed using the genome annotation system GenDB 2.0 [16]. In a second annotation step, all predicted ORFs were manually re-inspected to correct start codon and function assignments. Intergenic regions were checked for ORFs missed by the automatic annotation using the BLAST programs [46].

### Genomic comparisons

For comparative analyses, the annotated genome sequences of the following bacteria were imported into EDGAR [47]: *Actinosynnema mirum* DSM 43827 [GenBank:NC_013093], *Amycolatopsis mediterranei* U32 [GenBank:NC_014318], *Pseudonocardia dioxanivorans* CB1190 [GenBank:NC_015312, GenBank:NC_015313, GenBank:NC_015314], *Saccharopolyspora erythraea* NRRL 2338 [GenBank:NC_009142], *Saccharomonospora viridis* DSM 43017 [GenBank:NC_013159], and *Thermobispora bispora* DSM 43833 [GenBank:NC_014165]. The project is available as an open EDGAR project (http://edgar.cebitec.uni-bielefeld.de/cgi-bin/edgar_login.cgi?cookie_test=1&open=1) called EDGAR_BMC_Pseudonocardiaceae.

The gene content comparisons were done via a BLASTP analysis against the bactNOG subset of the eggNOG database [20]. Two genes were considered to be orthologs if the reciprocal BLASTP hit had a sequence identity of at least 40% and a coverage of at least 75%. For the phylogenetic analysis, all predicted CDS were compared against the RefSeq protein database [22] (from August 2011) using BLASTP. The species for each best hit (e-value cutoff 1e-10, hit must cover at least 75% of query and subject) was retrieved and counted.

All other genome comparisons were done using EDGAR, the PCA analyses were done using R (http://cran.r-project.org/).

### Analysis of secondary metabolite clusters

For the identification of secondary metabolite clusters the genome of *S. espanaensis* was scanned for homologues to known secondary metabolite synthases via BLAST search. These manual investigations were supported by antiSMASH [32]. A set of genes was considered to be a cluster, when there was at least one gene encoding a secondary metabolite synthase. Consequently, a locus possessing a gene with only a single domain, for example an A domain, was not considered to be a cluster. The boundaries of the clusters were defined by the last gene upstream and downstream of a secondary metabolite synthase with homology to a gene encoding a regulator, transporter or tailoring enzyme. In cases where this gene was part of a putative operon, the whole operon was included into the cluster. The modular organization of the type I polyketide and nonribosomal peptide megasynthases were determined using web tools [48,49]. The A domain specificities were investigated using NRPSpredictor2 [50,51].

### Nucleotide sequence accession numbers

The sequences reported here have been deposited in the EMBL database (accession no.: [EMBL:HE804045]). The locus tag prefix "BN6_" assigned by the ENA is replaced by "ses" throughout.

## Competing interests

The authors declare that they have no competing interests.

## Authors’ contributions

TS, annotated the saccharomicin gene cluster, coordinated and worked on the annotation of silent biosynthetic gene clusters and performed the comparison with the clusters of completely sequenced Pseudonocardiaceae. AAD worked on the initial stages of the genome finishing, performing primer design and gap closure. JB performed the EDGAR and PCA analyses. AG participated in the manual gene annotation. JK and AP were involved in coordination of the genome sequencing project. ML, worked on the identification and annotation of secondary metabolite clusters. RS created the sequencing libraries and performed the high-throughput sequencing steps. AB, assisted with the analysis of biosynthetic gene clusters. CR coordinated the initial phases of the genome sequencing and assembly, performed the final stages of genome finishing and polishing, and worked on the genome comparison. All authors read and approved of the final manuscript.

## Supplementary Material

Additional file 1 **Comparative analysis based on the general eggNOG functional categories.** The gene content comparisons were done via a BLASTP analysis against the bactNOG subset of the eggNOG database. Two genes were considered to be orthologs if the reciprocal BLASTP hit had a sequence identity of at least 40% and a coverage of at least 75%. Average and standard deviation were calculated for the relative numbers over all species excluding *S. espanaensis* and the cells were colored based on the significance level. Click here for file

Additional file 2 **Phylogenetic tree of the type and completely sequenced strains of the* Pseudonocardiaceae* family based on 16S rDNA sequences.** The 16S rDNA sequences of the relevant type strains were retrieved from RDP [52], with *Streptomyces avermitilis* added as an outgroup, and the 16S rDNA sequence of *A. mediterranei* U32 was taken from RefSeq. After alignment with the RDP pipeline, a phylogenetic tree was constructed using the Tree Builder of RDP. Completely sequenced strains are highlighted in bold type and color. Click here for file

Additional file 3 **Deduced functions of the ORFs located in the saccharomicin biosynthetic gene cluster from* S. espanaensis***. Click here for file

Additional file 4 **Gene clusters for secondary metabolites in* S. espanaensis*.** Click here for file

Additional file 5 **Deduced function of genes encoding nonribosomal peptide synthases in* S. espanaensis.*** Adenylation (A) domain numbering according to Stachelhaus *et al.*[53]. Substrates for A domains were determined using NRPSpredictor2 [50]. Their specificities are indicated when there is a nearest neighbor consistent in at least 8 residues and the prediction lies in the applicability domain of the model. Click here for file

Additional file 6 **Deduced function of genes encoding type I polyketide synthases in* S. espanaensis.*** Click here for file
