# Supplementary figures and images for: Complete genome sequence of Saccharothrix espanaensis DSM 44229T and comparison to the other completely sequenced Pseudonocardiaceae
Source: BMC Genomics. 2012 Sep 9;13:465. doi: 10.1186/1471-2164-13-465 (PMC3469384; doi:10.1186/1471-2164-13-465)

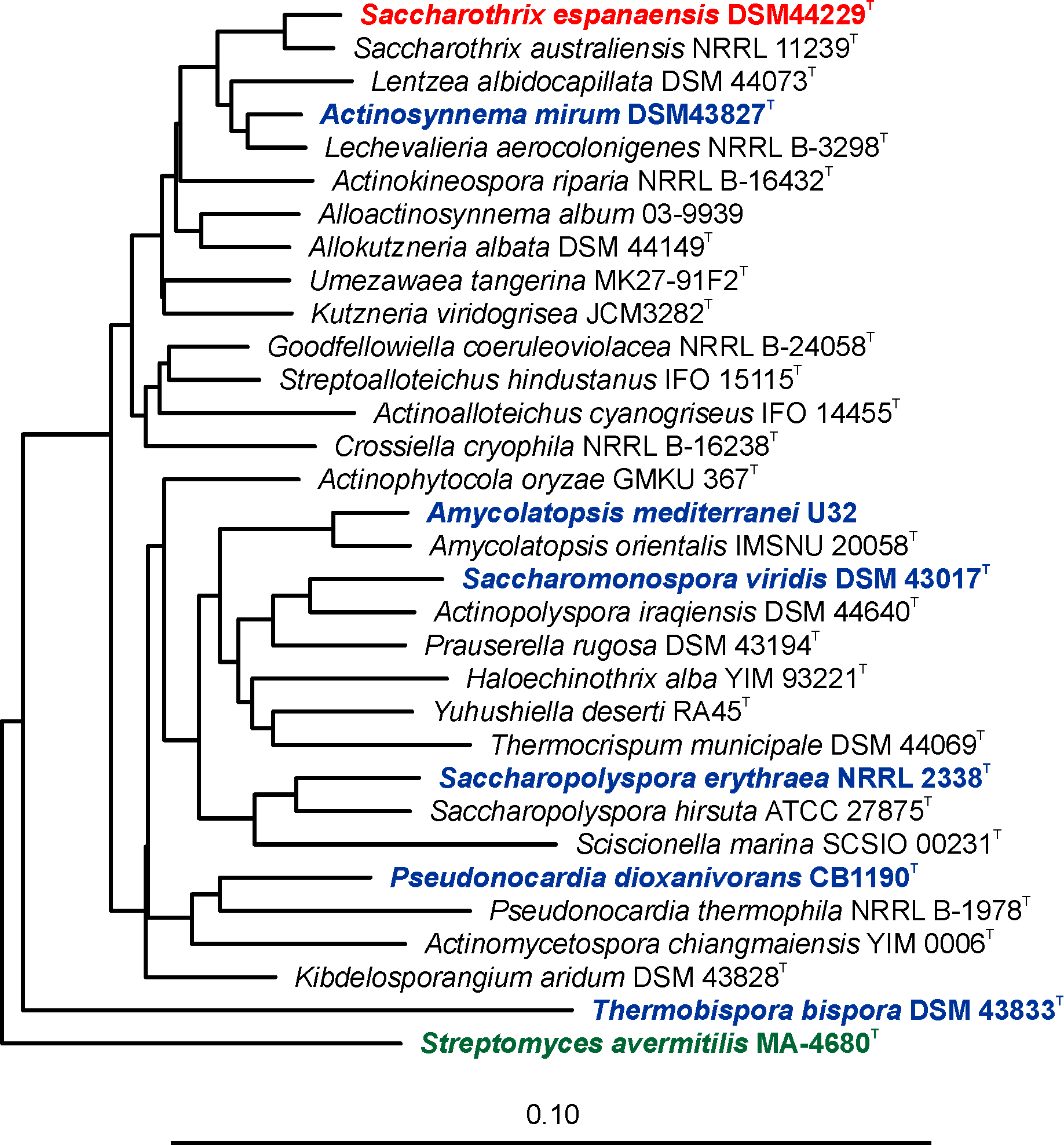

Supplement: Additional file 2 — Phylogenetic tree of the type and completely sequenced strains of the Pseudonocardiaceae family based on 16S rDNA sequences. The 16S rDNA sequences of the relevant type strains were retrieved from RDP [52], with Streptomyces avermitilis added as an outgroup, and the 16S rDNA sequence of A. mediterranei U32 was taken from RefSeq. After alignment with the RDP pipeline, a phylogenetic tree was constructed using the Tree Builder of RDP. Completely sequenced strains are highlighted in bold type and color. [file 1471-2164-13-465-S2.png]
