# Supplementary material for: Complete genome sequence of Saccharothrix espanaensis DSM 44229T and comparison to the other completely sequenced Pseudonocardiaceae
Source: BMC Genomics. 2012 Sep 9;13:465. doi: 10.1186/1471-2164-13-465 (PMC3469384; doi:10.1186/1471-2164-13-465)
Supplement: Additional file 3 — Deduced functions of the ORFs located in the saccharomicin biosynthetic gene cluster from S. espanaensis. [file 1471-2164-13-465-S3.doc]

**Additional file 3**

**Deduced functions of the ORFs located in the saccharomicin biosynthetic gene cluster from *S. espanaensis***

| **ORF** | **No. of amino acids** | **Deduced function** | **Protein homolog** | **% identity /  % similarity** | **Accession number** |
| --- | --- | --- | --- | --- | --- |
| ***sam1*** | 267 | transcriptional regulator | Psed_2412 [*Pseudonocardia dioxanivorans* CB1190] | 67/78 | AEA24616 |
| ***sam2*** | 377 | extracellular repeat protein | Geob_2007 [*Geobacter* sp. FRC-32] | 33/48 | YP_002537464 |
| ***sam3*** | 293 | transcriptional regulator | SSBG_00020 [*Streptomyces* sp. SPB74] | 44/54 | ZP_06825724 |
| ***sam4*** | 219 | hypothetical protein | FRAAL4627 [*Frankia alni* ACN14a] | 54/69 | YP_714812 |
| ***sam5*** | 512 | 4-coumarate 3-hydroxylase | function experimentally proved |  | ABC88666 |
| ***sam6*** | 290 | glucose-1-phosphate nucleotidyltransferase | sa14 [*Streptomyces aureofaciens*] | 67/82 | ACK77763 |
| ***sam7*** | 374 | Acetyl-coenzyme A synthetase | Ndas_4723 *Nocardiopsis dassonvillei* subsp. dassonvillei DSM 43111 | 47/58 | YP_003682615 |
| ***sam8*** | 510 | tyrosine ammonia-lyase | function experimentally proved |  | ABC88669 |
| ***sam9*** | 299 | putative S-adenosylmethionine-dependent methyltransferase | GQ274953.1 ORF10 [*Streptomyces platensis* subsp. rosaceus] | 45/60 | ACY01395 |
| ***sam10*** | 256 | hypothetical protein | MED193_14177 [*Roseobacter* sp. MED193] | 30/43 | ZP_01055406 |
| ***sam11*** | 424 | glycosyltransferase | desVII [*Streptomyces venezuelae*] | 45/58 | AAC68677 |
| ***sam12*** | 422 | glycosyltransferase | oleG2 [*Streptomyces antibioticus*] | 46/61 | CAA05642 |
| ***sam13*** | 425 | glycosyltransferase | Ndas_4716 [*Nocardiopsis dassonvillei* subsp. dassonvillei DSM 43111] | 46/61 | YP_003682608 |
| ***sam14*** | 420 | glycosyltransferase | Ndas_4707 [*Nocardiopsis dassonvillei* subsp. dassonvillei DSM 43111] | 44/56 | YP_003682599 |
| ***sam15*** | 419 | glycosyltransferase | Ndas_4716 [*Nocardiopsis dassonvillei* subsp. dassonvillei DSM 43111] | 50/63 | YP_003682608 |
| ***sam16*** | 424 | glycosyltransferase | Ndas_4716 [*Nocardiopsis dassonvillei* subsp. dassonvillei DSM 43111] | 46/60 | YP_003682608 |
| ***sam17*** | 420 | glycosyltransferase | Ndas_4716 [*Nocardiopsis dassonvillei* subsp. dassonvillei DSM 43111] | 48/60 | YP_003682608 |
| ***sam18*** | 420 | glycosyltransferase | Ndas_4727 [*Nocardiopsis dassonvillei* subsp. dassonvillei DSM 43111] | 50/61 | YP_003682619 |
| ***sam19*** | 427 | glycosyltransferase | Ndas_4716 [*Nocardiopsis dassonvillei* subsp. dassonvillei DSM 43111] | 47/61 | YP_003682608 |
| ***sam20*** | 422 | glycosyltransferase | desVII [*Streptomyces venezuelae*] | 45/61 | AAC68677 |
| ***sam21*** | 326 | NDP-4-keto-6-deoxyhexose 4-ketoreductase | jadV [*Streptomyces venezuelae* ATCC 10712] | 52/66 | AAL14256 |
| ***sam22*** | 330 | aldo/keto reductase | Tcur_2669 [*Thermomonospora curvata* DSM 43183] | 51/65 | YP_003300259 |
| ***sam23*** | 569 | membrane-flanked domain-containing protein | Xcel_2081 [*Xylanimonas cellulosilytica* DSM 15894] | 42/55 | YP_003326657 |
| ***sam24*** | 176 | membrane-flanked domain-containing protein | Snas_5070 [*Stackebrandtia nassauensis* DSM 44728] | 52/63 | YP_003513799 |
| ***sam25*** | 114 | hypothetical protein | Ndas_4706 [*Nocardiopsis dassonvillei* subsp. dassonvillei DSM 43111] | 51/64 | YP_003682598 |
| ***sam26*** | 188 | hypothetical protein | Ndas_4738 [*Nocardiopsis dassonvillei* subsp. dassonvillei DSM 43111] | 40/57 | YP_003682630 |
| ***sam27*** | 430 | L-threonine synthase | thrC1 [*Streptomyces bingchenggensis* BCW-1] | 54/67 | ADI04248 |
| ***sam28*** | 177 | hypothetical protein | Ndas_4730 [*Nocardiopsis dassonvillei* subsp. dassonvillei DSM 43111] | 38/57 | [YP_003682622](http://www.ncbi.nlm.nih.gov/protein/297563648?report=genbank&log$=prottop&blast_rank=1&RID=VJ6R4S0201P) |
| ***sam29*** | 140 | aspartate 1-decarboxylase | Franean1_0199 [*Frankia* sp. EAN1pec] | 58/78 | YP_001504572 |
| ***sam30*** | 369 | glutamine--scyllo-inositol transaminase | Ndas_4709 [*Nocardiopsis dassonvillei* subsp. dassonvillei DSM 43111] | 75/85 | YP_003682601 |
| ***sam31*** | 468 | NDP-hexose 2,3-dehydratase | ChlC3 [*Streptomyces antibioticus*] | 58/70 | AAZ77682 |
| ***sam32*** | 207 | dTDP-4-dehydrorhamnose 3,5-epimerase | SBI_06869 [*Streptomyces bingchenggensis* BCW-1] | 60/74 | ADI09989 |
| ***sam33*** | 331 | NDP-hexose 3-ketoreductase | KijD10 [*Actinomadura kijaniata*] | 56/71 | ACB46498 |
| ***sam34*** | 409 | NDP-hexose C-3 methyl transferase | dvaC [*Amycolatopsis balhimycina*] | 74/83 | CAC48364 |
| ***sam35*** | 175 | adenylylsulfate kinase | SghaA1_010100008098 [*Streptomyces ghanaensis* ATCC 14672] | 67/78 | ZP_04685125 |
| ***sam36*** | 798 | penicillin amidase | Rpic_4199 [*Ralstonia pickettii* 12J] | 51/65 | YP_001892723 |
| ***sam37*** | 419 | hypothetical protein | Ndas_1063 [*Nocardiopsis dassonvillei* subsp. dassonvillei DSM 43111] | 60/70 | YP_003679011 |
| ***sam38*** | 189 | RNA polymerase sigma factor | SACE_6625 [*Saccharopolyspora erythraea* NRRL 2338] | 66/75 | YP_001108716 |
