# Supplementary material for: Complete genome sequence of Saccharothrix espanaensis DSM 44229T and comparison to the other completely sequenced Pseudonocardiaceae
Source: BMC Genomics. 2012 Sep 9;13:465. doi: 10.1186/1471-2164-13-465 (PMC3469384; doi:10.1186/1471-2164-13-465)
Supplement: Additional file 4 — Gene clusters for secondary metabolites in S. espanaensis. [file 1471-2164-13-465-S4.doc]

**Additional file 4**

**Gene clusters for secondary metabolites in *S. espanaensis***

| **Cluster** | **Start** | **End** | **Genes** | **Length (bp)** | **Gene ID** |
| --- | --- | --- | --- | --- | --- |
| **Terpenes** | | | | | |
| **tpc 1 (geo)** | 1166889 | 1169105 | 1 | 2216 | *ses11180* |
| **tpc 2 (crt)** | 1706767 | 1712091 | 4 | 5324 | *ses16510*-*ses16540* |
| **tpc 3 (crt)** | 5680801 | 5688469 | 8 | 7668 | *ses50830*-*ses50900* |
| **tpc 4 (ptl)** | 6114314 | 6115375 | 1 | 1061 | *ses54650* |
| **tpc 5 (crt)** | 6295999 | 6297102 | 1 | 1103 | *ses56360* |
| **tpc 6 (2-MIB)** | 6630735 | 6631823 | 1 | 1088 | *ses59350* |
| **tpc 7 (crt)** | 6954768 | 6955850 | 1 | 1082 | *ses62220* |
| **Non-ribosomal peptides, polyketides and hybrids** | | | | | |
| **cluster 1** | 2349924 | 2370700 | 15 | 20776 | *ses22350*-*ses22490* |
| **cluster 2** | 2831236 | 2865923 | 16 | 34687 | *ses26880*-*ses27030* |
| **cluster 3** | 3453255 | 3504409 | 15 | 51154 | *ses32450*-*ses32590* |
| **cluster 4** | 3600126 | 3651122 | 30 | 50996 | *ses33420*-*ses33720* |
| **cluster 5** | 3831231 | 3881764 | 31 | 50533 | *ses35380*-*ses35690* |
| **cluster 6** | 4248529 | 4303665 | 44 | 55136 | *ses38940*-*ses39380* |
| **cluster 7** | 4972661 | 4999886 | 20 | 27225 | *ses45570*-*ses45780* |
| **cluster 8** | 5008350 | 5094309 | 41 | 85959 | *ses45860*-*ses46270* |
| **cluster 9** | 5186392 | 5271055 | 31 | 84663 | *ses47160*-*ses47500* |
| **cluster 10** | 5283255 | 5336168 | 33 | 52913 | *ses47640*-*ses47960* |
| **cluster 11** | 5367953 | 5424891 | 25 | 56938 | *ses48290*-*ses48530* |
| **cluster 12** | 5732076 | 5753813 | 11 | 21737 | *ses51300*-*ses51400* |
| **cluster 13** | 6124164 | 6177506 | 46 | 53342 | *ses54730*-*ses55180* |
| **cluster 14** | 6306803 | 6368410 | 45 | 61607 | *ses56460*-*ses56900* |
| **Others** | | | | | |
| **aminocyclitol** | 5883892 | 5891400 | 8 | 7508 | *ses52590*-*ses52660* |
| **lan 1** | 4529644 | 4536759 | 5 | 7115 | *ses41310*-*ses41350* |
| **lan 2** | 6044178 | 6076769 | 31 | 32591 | *ses54000*-*ses54300* |
| **mel** | 4916581 | 4917904 | 2 | 1323 | *ses45020*-*ses45030* |
| **sam** | 6549548 | 6596050 | 38 | 46502 | *ses58620*-*ses58990* |
| **26 clusters** |  |  | **505** | **822247** |  |

Abbreviations: geo, geosmin; crt, carotenoid; ptl, pentalenene; 2-MIB, methylisoborneol; lan, lantibiotic; mel, melanin; sam, saccharomicin.
