# Supplementary material for: Complete genome sequence of Saccharothrix espanaensis DSM 44229T and comparison to the other completely sequenced Pseudonocardiaceae
Source: BMC Genomics. 2012 Sep 9;13:465. doi: 10.1186/1471-2164-13-465 (PMC3469384; doi:10.1186/1471-2164-13-465)
Supplement: Additional file 5 — Deduced function of genes encoding nonribosomal peptide synthases in S. espanaensis. Adenylation (A) domain numbering according to Stachelhaus et al.[53]. Substrates for A domains were determined using NRPSpredictor2 [50]. Their specificities are indicated when there is a nearest neighbor consistent in at least 8 residues and the prediction lies in the applicability domain of the model. [file 1471-2164-13-465-S5.doc]

**Additional file 5**

**Table: Deduced function of genes encoding nonribosomal peptide synthases in *S. espanaensis***

| **Polypeptide** | **Gene** | **A domain residue** | | | | | | | | | | **Substrate** | **Module** |
| --- | --- | --- | --- | --- | --- | --- | --- | --- | --- | --- | --- | --- | --- |
|  |  | 235 | 236 | 239 | 278 | 299 | 301 | 322 | 330 | 331 | 517 |  |  |
| Nrps1 | *ses22480* | D | A | C | E | M | S | E | L | A | K | unknown | A-T-C |
| Nrps1-t2 | *ses22460* |  |  |  |  |  |  |  |  |  |  |  | T |
| Nrps1-a2 | *ses22430* | D | L | T | K | V | G | E | V | G | - | asn | A |
| Nrps1-t3 | *ses22390* |  |  |  |  |  |  |  |  |  |  |  | T |
| Nrps1-c2 | *ses22380* |  |  |  |  |  |  |  |  |  |  |  | C |
| Nrps1-a3 | *ses22370* | D | V | W | H | A | S | M | I | D | - | unknown | A |
| Nrps1-t4 | *ses22360* |  |  |  |  |  |  |  |  |  |  |  | T |
| Nrps2-1 | *ses26940* | D | A | E | D | V | G | T | V | V | - | unknown | C-A |
| Nrps2-2 | *ses26930* | D | V | W | H | I | S | L | V | D | K | ser | T-C-A-T |
|  |  | D | A | D | D | A | G | L | V | D | K | unknown | C-A-T-E |
|  |  | D | A | D | D | C | G | L | V | D | K | unknown | C-A-T |
|  |  | D | A | D | D | C | G | C | V | D | K | unknown | C-A-T-E |
|  |  | D | L | F | A | L | G | A | V | N | K | haorn | C-A-T-Te |
| Nrps3-1 | *ses32580* | D | A | L | L | I | G | A | V | V | K | val | A-T-C-T-E |
|  |  | D | F | W | N | I | G | M | V | H | K | thr | C-A-T |
| Nrps3-2 | *ses32570* | D | T | E | D | M | G | F | V | D | K | unknown | C-A-T-E |
|  |  | D | T | E | D | L | G | Y | V | D | K | unknown | C-A-T |
|  |  | D | T | E | D | M | G | F | V | D | K | unknown | C-A-T-E |
|  |  | D | F | W | N | I | G | M | V | H | K | thr | C-A-T |
|  |  | D | A | L | L | V | G | A | V | V | K | tyr | C-A-T-E |
| Nrps3-3 | *ses32470* | D | I | W | E | V | T | A | D | D | K | unknown | C-A-T-E |
|  |  | D | A | W | A | V | A | G | L | A | K | unknown | C-A-T |
|  |  | D | I | W | E | V | T | A | D | D | K | unknown | C-A-T |
|  |  | - | - | L | R | V | A | G | V | D | K | unknown | C-A-T-Te |
| Nrps5-1 | *ses35630* | D | L | L | W | L | G | G | T | F | K | val | C-A-T-? |
| Nrps5-2 | *ses35610* | D | V | V | Q | V | G | G | V | Y | K | unknown | C-A-T |
| Nrps5-3 | *ses35580* | D | I | L | Q | I | G | V | V | W | K | unknown | C-A-T |
| Nrps5-4 | *ses35560* | D | A | G | D | M | G | H | V | C | K | unknown | C-A-T-Red |
| Nrps5-a5 | *ses35450* | D | P | F | F | V | I | V | V | A | K | unknown | A |
| Nrps5-t5 | *ses35380* |  |  |  |  |  |  |  |  |  |  |  | T |
| Nrps6-c | *ses39030* |  |  |  |  |  |  |  |  |  |  |  | C |
| Nrps6 | *ses39040* | S | M | F | H | M | G | M | L | F | K | unknown | A-T |

| Nrps7 | *ses45580* | D | A | Y | F | L | G | G | T | F | K | val | A-T |
| --- | --- | --- | --- | --- | --- | --- | --- | --- | --- | --- | --- | --- | --- |
|  |  | D | A | Y | F | W | G | G | V | F | K | val | C-A-T-Red |
| Nrps8 | *ses46030* | D | F | W | S | V | G | M | V | H | K | thr | A-T |
| Nrpks8 | *ses46060* | D | L | F | N | F | S | L | V | W | K | cys | T-Cy-?-A-T-KS |
| Nrps10-1 | *ses47680* | D | V | W | H | F | S | L | V | D | K | ser | C-A-T |
|  |  | D | V | Q | F | I | S | Q | V | A | K | unknown | C-A-T-Te |
| Nrps10-2 | *ses47940* | D | V | W | N | V | A | M | V | H | K | ser | A-T |
| Nrps10-3 | *ses47910* | D | I | L | Q | V | G | V | I | W | K | gly | Cy/C-A-T-Te |
| Nrps10-4 | *ses47890* | A | A | F | - | V | T | L | V | A | K | unknown | C-A-T |
| Nrps10-a6 | *ses47870* | D | A | F | F | L | G | L | I | T | K | unknown | A |
| Nrps12-1 | *ses51320* | D | A | L | W | L | G | G | T | F | K | val | C-A-T |
|  |  | D | L | F | N | N | A | L | T | Y | K | ala | C-A-T |
| Nrps12-2 | *ses51330* |  |  |  |  |  |  |  |  |  |  |  | ?-C-T-C |
| Nrps13 | *ses55100* | D | L | Y | N | M | S | L | I | W | K | cys | C-A-T |
| Nrps14-1 | *ses56580* | D | I | L | A | - | - | - | V | L | - | unknown | C-A-? |
|  |  | - | - | A | Q | V | G | M | V | W | K | unknown | A-T-C |
| Nrps14-2 | *ses56610* | D | M | V | Q | F | G | L | V | Y | K | unknown | C-A-T |
|  |  | D | A | T | E | Q | S | Q | L | A | K | unknown | C-A-T |
| Nrps14-3 | *ses56790* | D | V | F | D | F | G | G | V | T | K | unknown | C-A-T |
| Nrps14-4 | *ses56830* | G | C | V | H | L | S | L | V | F | - | unknown | A-T |
| Nrps14-5 | *ses56850* | D | L | Y | N | I | A | S | V | W | K | unknown | C-A-T |

Abbreviations: A, adenylation; C, condensation; Cy, cyclization; E, epimerization; Red, reduction; T, thiolation; Te, thioesterase; ?, domain with novel function (domain of more than 450 aa with no similarity to known domains); KS, ketosynthase. haorn, delta-N-Acetyl-delta-N-hydroxyornithine.
