# Supplementary material for: Complete genome sequence of Saccharothrix espanaensis DSM 44229T and comparison to the other completely sequenced Pseudonocardiaceae
Source: BMC Genomics. 2012 Sep 9;13:465. doi: 10.1186/1471-2164-13-465 (PMC3469384; doi:10.1186/1471-2164-13-465)
Supplement: Additional file 6 — Deduced function of genes encoding type I polyketide synthases in S. espanaensis. [file 1471-2164-13-465-S6.doc]

**Additional file 6**

**Table: Deduced function of genes encoding type I polyketide synthases in *S. espanaensis***

| **Polypeptide** | **Gene** | **Module** | **Deduced function** |
| --- | --- | --- | --- |
| **Pks4-1** | *ses33510* | module 1 | KS-AT-DH-ER-KR-ACP |
| **Pks4-2** | *ses33620* | module 2 | KS-AT-ACP |
|  |  |  | KR-ACP |
| **Pks4-3** | *ses33690* | module 3 | KS-AT-DH-?-ER-KR-ACP |
| **Pks5** | *ses35390* | load | KS-ACP |
| **Pks6-1** | *ses39100* | module 1 | KS-AT-DH-KR-ACP |
| **Pks6-2** | *ses39290* | module 2 | KS-AT-ACP-KR-DH |
| **Nrpks8** | *ses46060* | module 2 | T-Cy-?-A-T-KS |
| **Pks8-1** | *ses46180* | load | ACP |
|  |  | module 1 | KS-KR-ACP |
|  |  | module 2 | KS |
| **Pks8-2** | *ses46170* | module 2 | DH-ACP |
|  |  |  | KR |
|  |  | module 3 | KS-ACP |
|  |  | module 4 | KS-DH-KR-ACP |
|  |  | module 5 | KS-KR-ACP |
|  |  | module 6 | KS-ACP-ACP |
| **Pks8-3** | *ses46000* |  | KS |
| **Pks8-4** | *ses45860* | module 7 | KS-AT-ACP-KR |
| **Pks8-5** | *ses45870* |  | KS-AT |
| **Pks9-1** | *ses47260* | load | KS-ACP |
|  |  | module 1 | KS-AT-DH-KR-ACP |
|  |  | module 2 | KS-AT-DH-KR-ACP |
|  |  | module 3 | KS-AT-DH-KR-ACP |
| **Pks9-2** | *ses47240* | module 4 | KS-AT-KR-ACP |
| **Pks9-3** | *ses47230* | module 5 | KS-AT-KR-ACP |
|  |  | release | TRed |
| **Pks9-4** | *ses47420* | module 1 | KS-AT-ACP |
|  |  | module 2 | KS-AT-DH-KR-ACP |
| **Pks9-5** | *ses47410* | module 3 | KS-AT-KR-ACP |
| **Pks9-6** | *ses47400* | module 4 | KS-AT-DH-KR-ACP |
|  |  |  | KS-AT |
| **Pks9-7** | *ses47370* | module 5 | KS-AT-DH-KR-ACP |
|  |  | release | TE |
| **Pks10** | *ses47930* | module 1 | KS-AT-?-KR-ACP |
| **Pks11-1** | *ses48330* | module 1 | KS-AT-ACP |
|  |  | module 2 | KS-AT-DH-KR-ACP |
|  |  | module 3 | KS-AT-DH-KR-ACP |
| **Pks11-2** | *ses48320* | module 4 | KS-AT-DH-KR-ACP |
|  |  | module 5 | KS-AT-DH-ACP |
| **Pks11-3** | *ses48310* | module 6 | KS-AT-DH-KR-ACP |
|  |  | release | TRed |
| **Pks13** | *ses55110* | module 1 | KS-AT-DH-KR-ACP |
| **Pks14** | *ses56590* | module 1 | KS-AT-ACP |
|  |  |  | KR-ACP |

Abbreviations: ACP, acyl carrier protein; AT, acyltransferase; Cy, cylization; DH, dehydratase; ER, enoylreductase; KR, ketoreductase; KS, ketosynthase; TE, thioesterase; TRed, thioester reductase; ?, domain with novel function (domain of more than 450 aa with no similarity to known domains); A, adenylation; T, thiolation.
